# Machine Learning in Medical Emergencies: a Systematic Review and Analysis

**DOI:** 10.1007/s10916-021-01762-3

**Published:** 2021-08-18

**Authors:** Inés Robles Mendo, Gonçalo Marques, Isabel de la Torre Díez, Miguel López-Coronado, Francisco Martín-Rodríguez

**Affiliations:** 1 Department of Signal Theory and Communications and Telematics Engineering, University of Valladolid, Paseo de Belén, 15, 47.011 Valladolid, Spain; 2 Advanced Clinical Simulation Center. Faculty of Medicine, University of Valladolid, Avda. Ramón Y Cajal, 7, 47.005 Valladolid, Spain; 3 Polytechnic of Coimbra, ESTGOH, Rua General Santos Costa, 3400-124 Oliveira do Hospital, Portugal

**Keywords:** Machine learning, Health emergencies, Emergency medicine, Mobile applications

## Abstract

Despite the increasing demand for artificial intelligence research in medicine, the functionalities of his methods in health emergency remain unclear. Therefore, the authors have conducted this systematic review and a global overview study which aims to identify, analyse, and evaluate the research available on different platforms, and its implementations in healthcare emergencies. The methodology applied for the identification and selection of the scientific studies and the different applications consist of two methods. On the one hand, the PRISMA methodology was carried out in *Google Scholar*, *IEEE Xplore*, *PubMed ScienceDirect,* and *Scopus*. On the other hand, a review of commercial applications found in the best-known commercial platforms (Android and iOS). A total of 20 studies were included in this review. Most of the included studies were of clinical decisions (*n* = 4, 20%) or medical services or emergency services (*n* = 4, 20%). Only 2 were focused on m-health (*n* = 2, 10%). On the other hand, 12 apps were chosen for full testing on different devices. These apps dealt with pre-hospital medical care (*n* = 3, 25%) or clinical decision support (*n* = 3, 25%). In total, half of these apps are based on machine learning based on natural language processing. Machine learning is increasingly applicable to healthcare and offers solutions to improve the efficiency and quality of healthcare. With the emergence of mobile health devices and applications that can use data and assess a patient's real-time health, machine learning is a growing trend in the healthcare industry.

## Introduction

Nowadays, people are familiar with artificial intelligence and machine learning. These terms are beginning to be present in all facets of the world, whether consciously or unconsciously, as they are linked to computer processes and automated artificial intelligence systems [1].

Artificial intelligence is a field that is composed of computer science methods and reliable datasets. The objective of artificial intelligence is to provide novel and effective methods for problem-solving. It also encompasses sub-fields of machine learning and deep learning, which are frequently mentioned in conjunction with artificial intelligence. These disciplines are comprised of AI algorithms that seek to create expert systems that make predictions or classifications based on input data. The complexity of artificial intelligence systems ranges from simple methods direction for specific tasks to abstract methods that aim to imitate human intelligence using computers.

The applications in everyday life with artificial intelligence and machine learning systems are numerous, and the implementation of these systems, and in particular their growth, is due to the increase in the availability of data that our society is experiencing [2]. These types of artificial intelligence applications take advantage of the large volume of data to extract as much information as possible from it and help to elaborate tasks that require human intelligence, currently transferred to computer systems [3, 4].

**Fig. 1:**
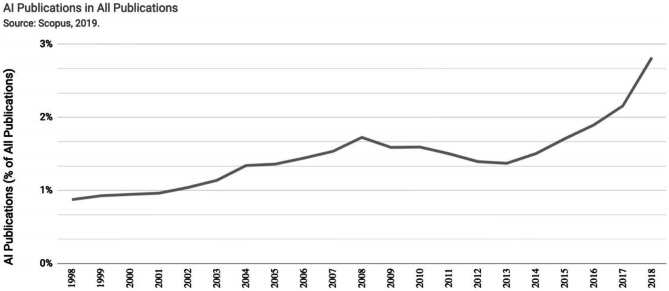
Growth of IA-related articles between 1998 and 2019 [7]

The field of artificial intelligence is a set of algorithms that simulates human intelligence embedded in machines, to make them have the same capabilities as human beings [5]. In recent years, interest in AI research has grown exponentially, leading to the development of numerous new applications in a multitude of fields [6]. Figure 1 shows how the number of articles related to this topic is growing more and more as the years go by. From less than 1% in 1998 to almost 3% in 2018 [7].

There are different fields of AI learning. Machine learning and deep learning differ from each other in the way they analyse and manipulate data and are used to train machines to mimic human intelligence and behaviour [8].

Machine learning is a critical field of artificial intelligence that includes statistical methods and algorithms to make predictions using existent data to support the learning process. On the one hand, deep learning is a subfield of machine learning. Deep learning enables the automation of feature extraction to eliminate some processes that have been typically done by human intervention and make it possible to use a high volume of information. On the other hand, these processes need to be manually implemented when using machine learning algorithms.

Machine Learning has been gaining popularity in recent years. It is based on using user-defined algorithms to detect patterns in massive data and make predictions, all autonomously [9]. The purpose of this field of AI is to perform specific tasks without having to be programmed to do so and without having to depend on a rule-based system [10]. On the other hand, deep learning uses artificial neural networks (ANNs) to carry out the ML process, mentioned above, and learn how neurons in the brain work.

The conclusion is that as you go up the level, this information becomes more complex. This learning is called deep learning because of the layered representation model and requires longer periods than ML, due to the multitude of layers of complex computations it must solve [11].

According to the definition provided by the American College of Emergency Physicians, emergency medicine is a medical focused on the diagnosis and treatment of illness or injury. It has a fundamental role to play in society by receiving patients seeking urgent medical care [12]. The practice performed by EM physicians includes the initial assessment, diagnosis, treatment, coordination of care among different physicians, and stabilisation of cases of varying degrees of acuity for any given patient [13].

The use of machine learning and deep learning can help to assess data collected over the past years and provide the information needed to improve emergency medical processes [14]. It can assist in research and medical trials based, for example, on clinical image analysis and classification tasks, thereby automating the process and helping to reduce the workload of medical staff, thus avoiding possible human fatigue that can lead to errors in the important area of healthcare.

Moreover, emergency medicine has been a major focus for study and solution development with the advent of artificial intelligence. Technological advances have brought various tools that have great potential to improve processes and will improve the operational efficiency and quality of healthcare service delivery [14].

There are several studies that support interventions based on artificial intelligence and can match or surpass the expertise of physicians. On the other hand, there are also a multitude of mobile health apps for mobile devices, which offer good options for patient progression, as well as providing patient education materials, receiving personalised guidance and support, data retrieval, and use of self-management interventions should the user need them [15].

Furthermore, this study is necessary to know the current situation in different search platforms and to find out the different utilities that these computer systems are capable of. Currently, proposed methods can perform tasks that would normally require human intelligence, oriented towards the field of medicine.

## Methodology

The methodology followed was based on carrying out two different types of systematic reviews:

The first was a review of the literature in articles extracted from different open access scientific search engines for the University of Valladolid. The second review was carried out on different commercial application platforms.

Both reviews were carried out until the end of April 2021, therefore, all articles and applications published later have not been considered in the analysis of the following work.

### Literature review

Before beginning to detail the characteristics of the PRISMA-ScR protocol, the articles to be selected must first be obtained, that is why we proceed to specify the search strategy used to find a series of articles. These 3 procedures of the search strategy were carried out successively in each one of the search engines. In this literature review, articles were retrieved from different academic search systems: *Google Scholar*, *IEEE Xplore*, *PubMed*, *ScienceDirect* and *Scopus*. The authors have selected these databases for two main points. On the one hand, they cover most of the scientific information in fields such as engineering or telemedicine. On the other hand, these databases have been used by several recent systematic reviews on health informatics [16–20].

In each of these databases, an advanced search is carried out by combining appropriate words and/or expressions, making use of AND and OR connectors. To carry out the research, specifically the Machine Learning analysis in the field of health, the following keywords have been mainly used in combination with each other, although there may be many more combinations, depending on the criteria of each user: (“All Metadata”: machine) AND (“All Metadata”: learning) OR (“All Metadata”: AI) AND (“All Metadata”: health*) AND (“All Metadata”: emergenc*) OR (“All Metadata”: urgenc*).

To minimise this large number of searches and to assist in the selection of suitable items, various filters are applied. The parameters set were as follows:

- The date of publication of the article must be between 2011 and the present day.
- The language must be English and Spanish only.
- The document type criterion. Only documents that are research or systematic review articles will be retained, as they gather information from the most relevant sources.

**Appendix A** shows schematically the procedure used in the search strategy. The authors used the PRISMA-ScR Protocol. This protocol has 4 phases necessary to perform such a state-of-the-art study. These phases will be carried out in all search systems until the existing results are finalised [21] Fig. 10.

Identification phase: Articles were discarded if, despite including the keywords and filters, they did not go into the subject matter of this research work in depth. To carry out the first round of discarding, the articles obtained were sorted in order of relevance and the title was read. If the article does not have the expected title, it will be discarded.

Selection phase: The documents obtained in the previous phase were screened for duplicate articles. Moreover, the summary of each of the documents is consulted and discarded in case it is not potentially what is expected.

Eligibility phase: In this stage, the article is read individually and in its entirety. Insufficiently informative articles will be discarded. The opinion of the author/authors and their conclusions will be considered, as well as the number of citations the article has.

Inclusion phase: Finally, articles that have not been discarded in the previous phase will be included in this review.

The risk of bias assessment points out the transparency of the results provided by the studies included in a systematic review. Several systematic reviews on related topics available in the literature that has adopted PRISMA methodology do not include the risk of bias [22, 23]. Consequently, this study does not list the risk of bias. Nevertheless, the authors highlight this limitation.

### Review of commercial mobile applications

A review of currently available mobile applications is also carried out, to which different search strategies are applied. The collection of mobile apps (mHealth) was carried out in the most popular search systems of the smartphone brands. In this case, the two main and most used app shops have been chosen: App Store used on iOS devices and Google Play used on Android devices [9].

Search terms: The first procedure is similar to the previous review. A set of keywords is chosen that fit the desired search. The set of words was as follows: “mHealth”, “eHealth”, “Machine learning and emergencies”, “Machine learning and health”, “AI and health”, “Al and medicine”, “mHealth and CoreML (for App Store search)” y “mHealth y MLKit (for Google Play search)”. To select the relevant mobile applications; several criteria have been followed:

- The main categories will be "medicine" and "health and wellness". Other apps focused in other categories will be discarded.
- Apps that have a cost will be discarded.
- The rating must be equal to or higher than 2 stars.
- The language of the applications must be English and/or Spanish.

The description of each application is read and then downloaded to test each application individually and in full. Apps with insufficient information will be discarded [15]. For applications from the Google Play platform, a Realme 7 5G with Android 10 operating system was used, for applications from the App Store platform, an iPhone 7 with iOS 14.5 operating system (latest update until April 2021) was used.

**Appendix B** shows the flowchart describing the above-mentioned procedure Fig.11.

## Results

In total, 20 potential information articles in the literature review and 12 mobile applications in the review of commercial applications were chosen for the analysis.

### Literature review

First, applying the corresponding keywords and filters, a total of 1,583 articles were retrieved through searches in the identified bibliographic databases, as shown in the following Table 1.

**Table 1:** Number of results obtained in search engines

Academic Search Systems	Search terms	Search filtering
Google Scholar	+1.600.000	29
IEEE Xplore	126.218	579
PubMed	269.704	41
Science Direct	2.061	320
Scopus	365	34
	**Total:**	**1.583**

Different criteria were applied to the articles found, discarding 1,478 records whose titles did not meet expectations during the Identification phase. In this selection phase, the summary of each of the 105 documents was consulted, and 74 documents were discarded since, in the opinion of the authors, they were not potentially what was expected.

In total, 31 articles were found for content analysis during the eligibility stage and 11 of them were subsequently eliminated because the information they contained did not focus on healthcare emergencies. Finally, after eliminating several articles with the different criteria mentioned above, 20 articles are included in this systematic review.

The screening process of the included results has been done at least by two reviewers independently. This process has two main stages. On the one hand, the reviewers have read the title and abstract of each study. On the other hand, the reviewers have analysed all the manuscript content. The study is included only with the common consensus of the reviewers.

**Fig. 2:**
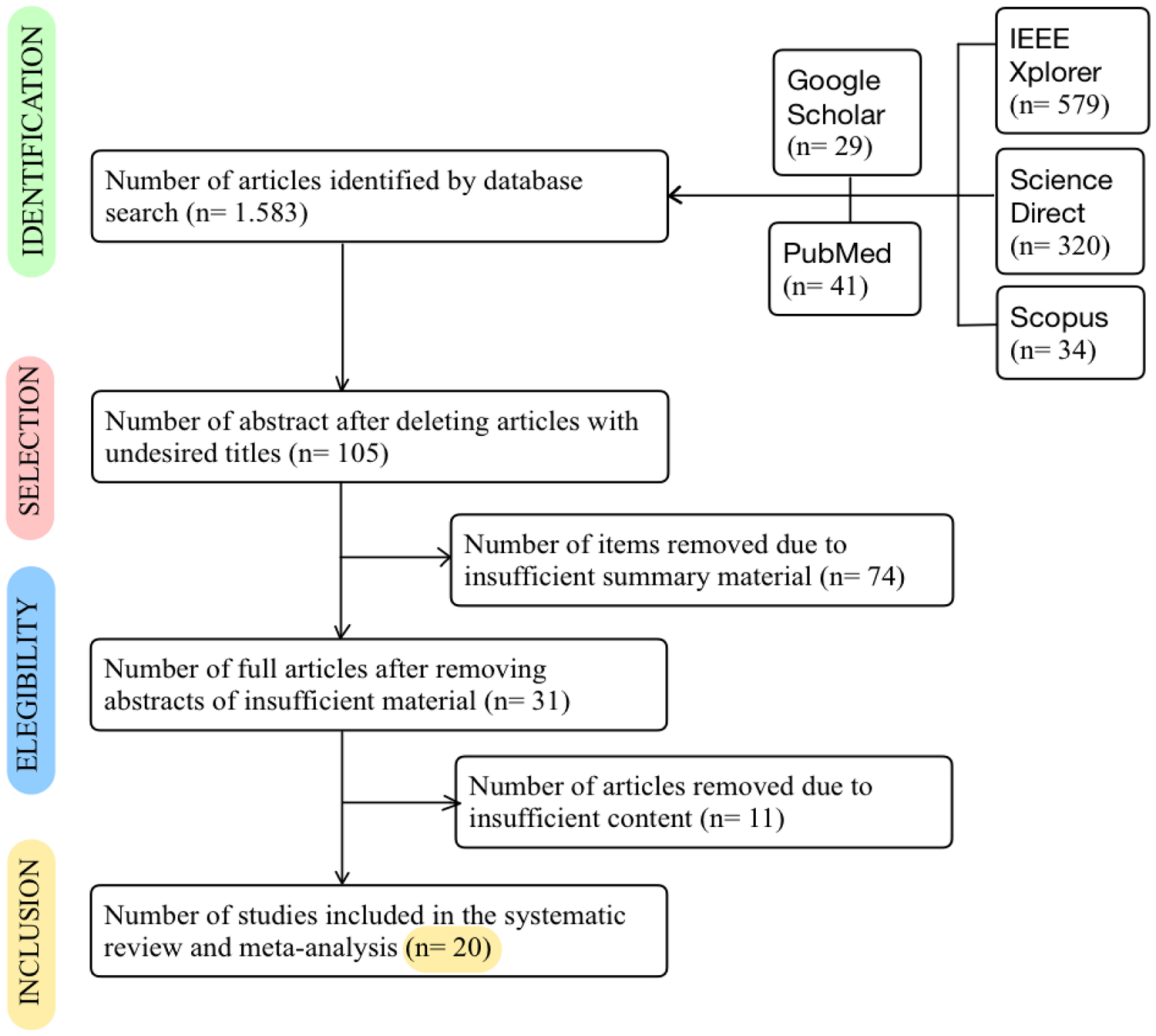
Numeric flow diagram of the PRISMA-ScR protocol algorithm

Figure 2 shows a flow diagram describing the PRISMA-ScR protocol algorithm with numbers.

**Fig. 3:**
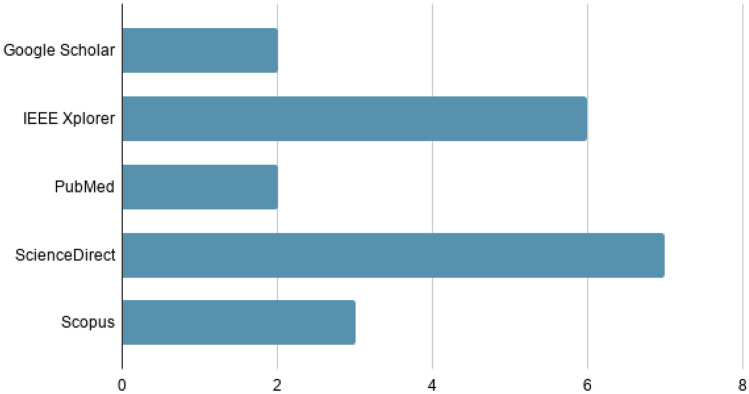
Number of results in the literature review search

Figure 3 shows the number of articles from each search engine included in this work.

**Fig. 4:**
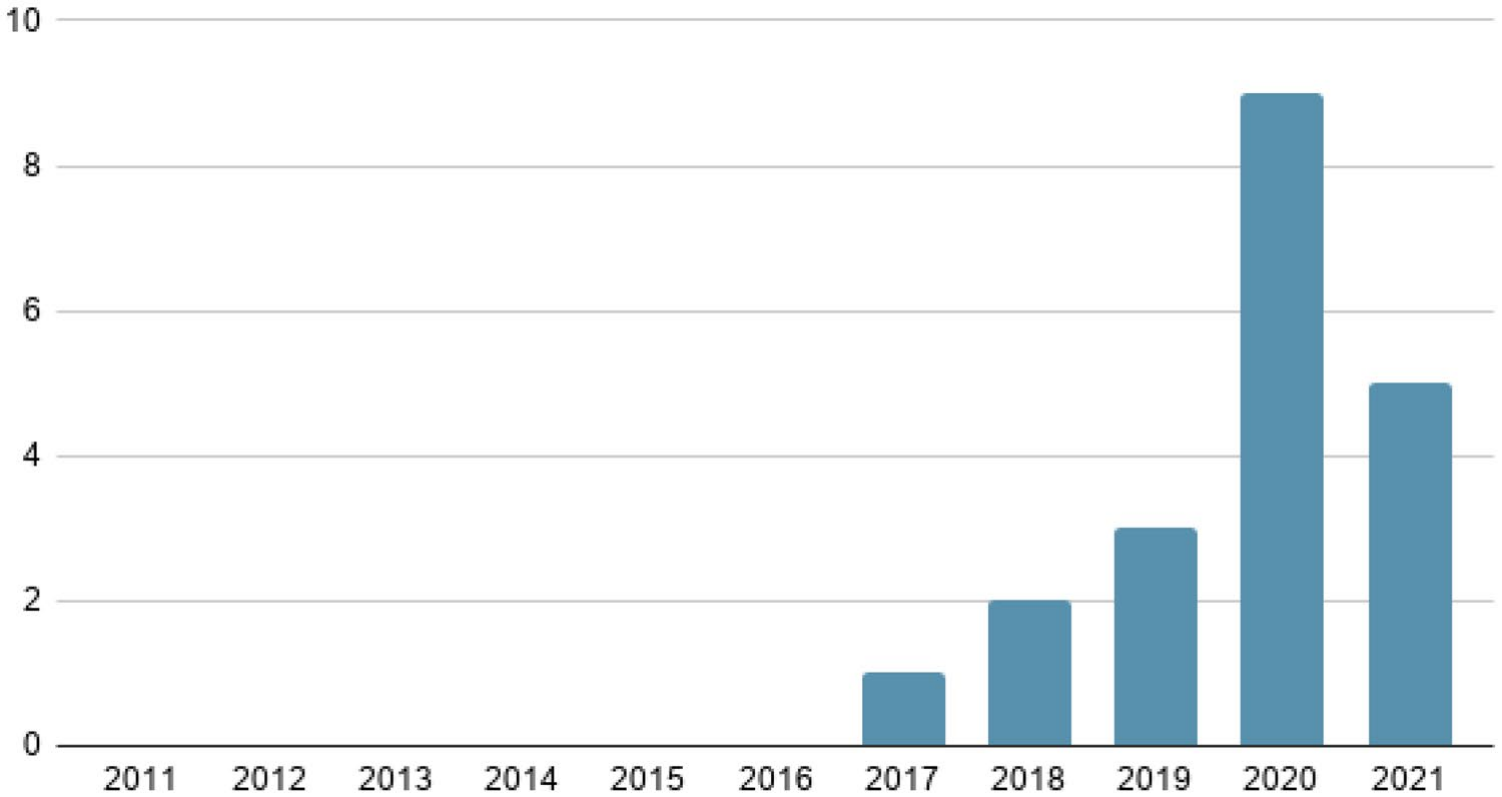
Number of articles published in the last 10 years

The Science Direct search engine is the platform where most articles of interest have been selected, followed by IEEE Xplore. Figure 4 shows the number of articles published each year over the last 10 years.

The year 2020 is notable for having 9 articles with information of interest compared to previous years. This is mainly due to research into COVID-19, considered a global health emergency disease, whose need for detection and treatment through machine learning, among others, is of vital importance at present. The included studies are divided into 7 major groups according to the content covered in each of them, as shown in Table 2. These categories have been defined based on the included studies and under the supervision and medical background of the last author. Group 1 includes three studies that focus on methods or systems to support pre-hospital medical care and disease screening. Group 2 includes four works that focus on machine learning-based solutions for clinical decision support. Group 3 presents three research studies that focus on the current pandemic scenario of COVID-19. Group 4 (Emergency medicine) includes 3 review studies focused on health informatics solutions for medical scenarios that require immediate medical attention. Group 5 includes three works that focus on services for enhanced medical scenarios. Group 6 includes two research papers that deal with the applications of mobile computing technologies. Finally, the remaining one study has been categorized as others since cannot be included in any of the before mentioned categories.

**Table 2:** Categorization of literature

Group	Total	References	Percentage
Pre-hospital medical care and disease screening	3	[24–26]	15%
Clinical decisions	4	[27–30]	20%
COVID-19 screening, and management	3	[31–33]	15%
Emergency medicine (EM)	3	[34–36]	15%
Medical services and/or emergency services	4	[37–40]	20%
M-Health	2	[41, 42]	10%
Others	1	[43]	5%

**Fig. 5:**
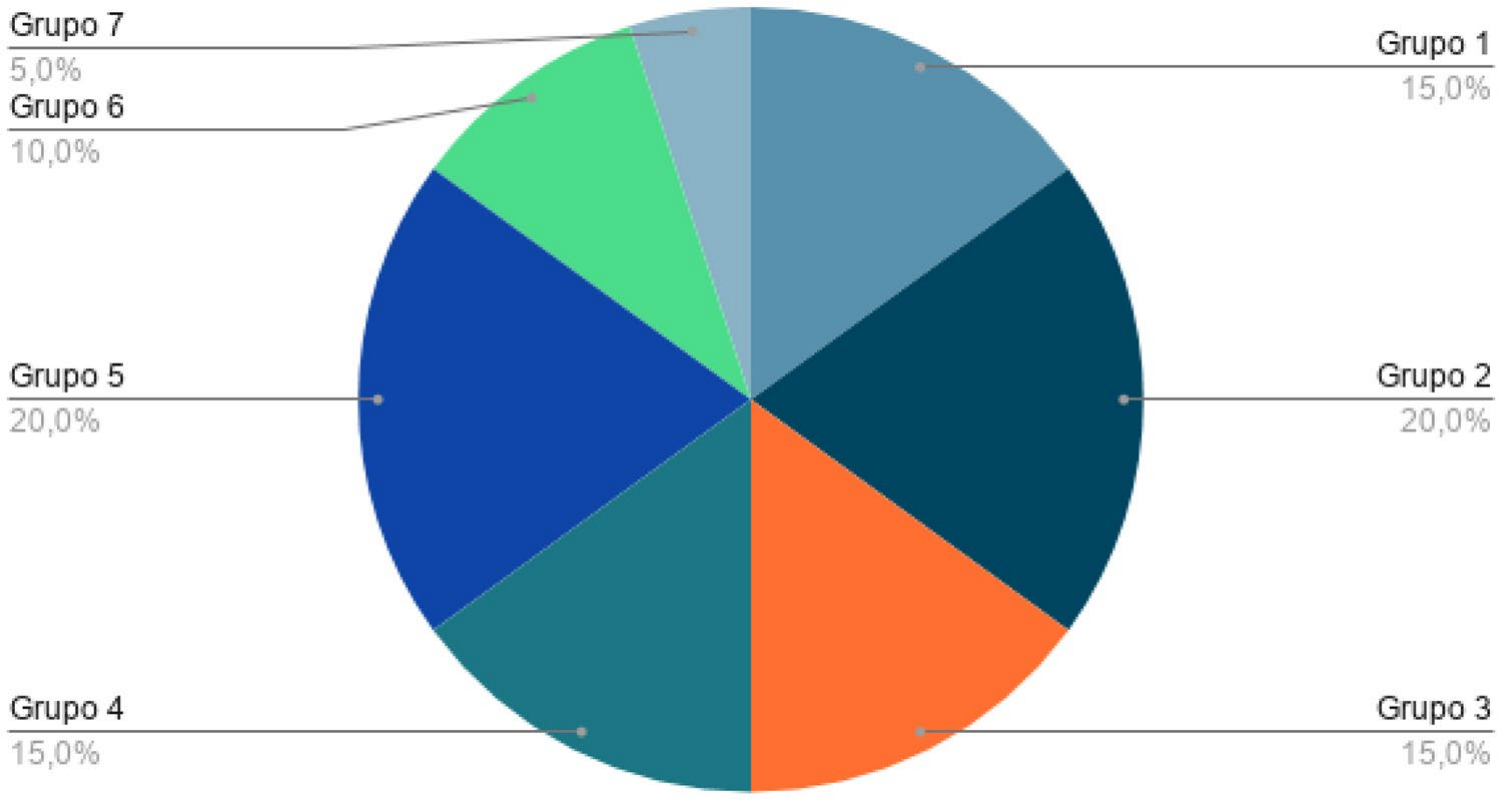
Categorization of literature in groups according to the content covered

Figure 5 represents the distribution of the selected literature concerning the group information represented in Table 2.

### Analysis

Table 3 presents the title, date, authors, and main contributions addressed in these articles [14–33].

**Table 3:** Characteristics of the selected items

Title and date	Author(s)	Main Contribution	Category
An accurate and dynamic predictive model for a smart M-Health system using machine learning [42] October 2020	Naseer Qreshi K, Din S., & Jeon G	This model is divided into data collection, data pre-processing, data partitioning, learning algorithm and the decision making for which it has been trained	M-Health
Applications of machine learning and artificial intelligence for Covid-19 (SARS-CoV-2) pandemic: A review [32] October 2020	Lalmuanawma, S., Hussain, J., & Chhakchhuak, L	This report reviews existing information on the application of ML and AI to address the COVID-19 pandemic	COVID-19 screening, and management
Applications of Machine Learning Approaches in Emergency Medicine [36] June 2019	Shafaf N., & Malek H	This paper aims to compile and evaluate the existing studies in recent years on AI in EM, which can be categorised into different groups	Emergency medicine (EM)
Architecture of Smart Health Care System Using Artificial Intelligence [25] 2020	Kamruzzaman M. M	It is concluded that AI- or ML-based healthcare offers a multitude of improvements for the health sector	Pre-hospital medical care and disease screening
Artificial Intelligence and Machine Learning Applications in Musculoskeletal Imaging [43] February 2019	Enamandram S., Sandhu E., Bao H.Do, Reicher J., & Beaulieu CF	This article describes the key applications of supervised and unsupervised ML in musculoskeletal medicine. Such as diagnostic imaging, patient measurement data and clinical decision support	Others
Artificial Intelligence and Machine Learning in Emergency Medicine [34] July 2018	Stewart, J., Sprivulis, P., & Dwivedi, G	This article studies and conducts a research analysis of AI and ML in EM Finally, it is emphasised that, despite limitations, AI and its subfields are very useful as they can solve problems in a wide range of clinical domains	Emergency medicine (EM)
Artificial Intelligence for the Future Radiology Diagnostic Service [29] January 2021	Mun, S.K. Wong, K.H.,Lo, S.-C.B.,Li, Y., & Bayarsaikhan, S	In this chapter, artificial intelligence (AI) is explored along future lines in diagnostic radiology Three avenues are proposed for the important role of AI in radiology beyond current capabilities	Clinical decisions
Automatic Clinical Procedure Detection for Emergency Services [28] July 2019	Heard, J., Paris, R. A., Scully, D., McNaughton, C., Ehrenfeld, J. M., Coco, J., Fabbri, D., Bodenheimer, B., & Adams, J. A	A system based on human activity recognition algorithms to accurately recognise clinical processes and send data of these processes without the presence of the physician is evaluated	Clinical decisions
Classification of hospital admissions into emergency and elective care: a machine learning approach [39] November 2017	Krämer, J., Schreyögg, J., & Busse, R	This article focuses on the classification of hospital admissions in emergency care with a focus on ML	Medical services and/or emergency services
Clinician involvement in research on machine learning-based predictive clinical decision support for the hospital setting [30] March 2021	Schwartz, J. M., Moy, A. J., Rossetti, S. C., Elhadad, N., & Cato, K. D	This article describes the involvement of clinicians in the development, evaluation and implementation of clinical decision support systems that use ML and analyse electronic medical record data to assist clinicians in their diagnosis and treatment, as well as in decision making	Clinical decisions
Development, evaluation, and validation of machine learning models for COVID-19 detection based on routine blood tests [31] October 2020	Cabitza, F., Campagner, A.,Ferrari, D., Di Resta, C., Ceriotti, D., Sabetta, E., Colombini, A., De Vecchi, E., Banfi, G.,Locatelli, M., & Carobene, A	This paper studies the development and evaluation of machine learning models for the detection of COVID-19 based on blood tests The methodology carried out was to train 3 different datasets to develop different predictive models	COVID-19 screening, and management
Fall Detection for Elderly People using Machine Learning [37] July 2020	Badgujar S., & Pillai AS	This paper presents a fall detection system based on wearable sensors that are suitable for elderly people	Medical services and/or emergency services
IoT based healthcare monitoring system using 5G communication and Machine learning models [26] January 2021	Paramita, S., Bebartta, H. N. D., & Pattanayak, P	This smart system for patients by implanting wireless sensors in the body collects different vital aspects such as heart rate, blood pressure, etc	Pre-hospital medical care and disease screening
Machine learning and artificial intelligence in the service of medicine: Necessity or potentiality? [40] November 2020	Alsuliman, T., Humaidan, D., & Sliman, L	This article arises because of the global trend towards digitisation of the healthcare system and how the need for it affects this area	Medical services and/or emergency services
Machine Learning for Predicting Emergency Incidents that Need an Air-ambulance [38] July 2020	Nuntalid N., & Richards D	The main objective of this article is to develop a real-time report to help the emergency medical service improve patient outcomes	Medical services and/or emergency services
Machine Learning-Based Early Warning Systems for Clinical Deterioration: Systematic Scoping Review [27] February 2021	Muralitharan, S., Nelson, W., Di, S., McGillion, M., Devereaux, P., Barr, N. G., & Petch, J	The results obtained in this article are based on a systematic scoping review following the PRISMA-ScR model and conclude that the impact on ML-based early warning systems could be significant for clinicians and patients because of the decrease in false alerts and the increase in early detection	Clinical decisions
Machine Learning-based Risk of Hospital Readmissions: Predicting Acute Readmissions within 30 Days of Discharge [24] 2019	Baig, M. M., Hua, N., Zhang, E., Robinson, R., Armstrong, D., Whittaker, R., Robinson, T., Mirza, F., & Ullah, E	The proposed predictive model works better than other admission risk models. However, to further strengthen risk prediction and its clinical impact, the addition of non-clinical data such as social support is proposed as a future line of research	Pre-hospital medical care and disease screening
Review on machine and deep learning models for the detection and prediction of Coronavirus [33] June 2020	Ahmad W., Salehi Preety B., & Gaurav G	Solutions are proposed through AI by performing a scoping review following the PRISMA-ScR model The research concludes that so far there is no effective drug for the treatment of patients with COVID-19, but early detection or prediction of coronavirus cases may be possible with these predictive models	COVID-19 screening, and management
Role of machine learning in medical research: A survey [35] May 2020	Garg A., & Mago V	Different concepts of ML and DL and their possible medical application are studied and analysed	Emergency medicine (EM)
SaveMe: A Crime Deterrent Personal Safety Android App with a Bluetooth Connected Hardware Switch [41] August 2018	Tripti, N. F., Farhad, A., Iqbal, W., & Zaman, H. U	This application consists of a switch connected to the smartphone via Bluetooth that is pressed to alert the emergency contact of the victim in question of danger	M-Health

### Performance in the review of commercial mobile applications

Firstly, several searches were conducted on the commercial platforms. The desired keywords were applied and a total of 3,089 applications were retrieved. On the one hand, 2084 results are available in App Store and 1005 in Google Play.

Once the results of the previous table had been obtained, various filters were applied to narrow down the results, resulting in 201 applications, of which the description was read to find the most appropriate thematic applications. A total of 168 were discarded, resulting in 33 applications, which will be subjected to a final criterion. Finally, the 21 less generic applications that did not cover the treatment and/or care of different health emergency diseases were eliminated. Applications with similar functionality to others were also removed, resulting in a total of 12 applications.

**Fig. 6:**
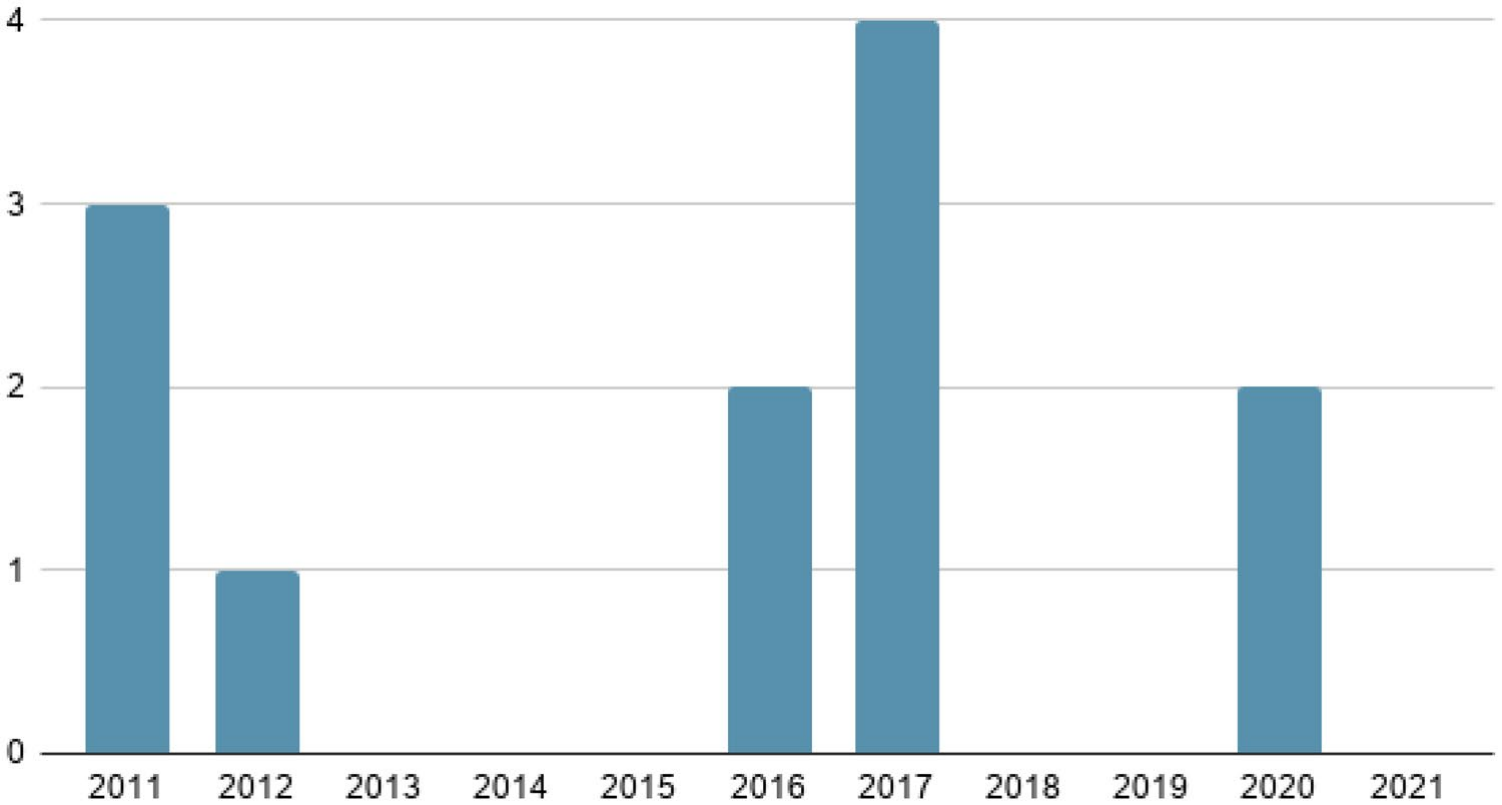
Number of apps published in the last 10 years

Figure 6 also shows graphically the number of applications launched in the different years.

**Fig. 7:**
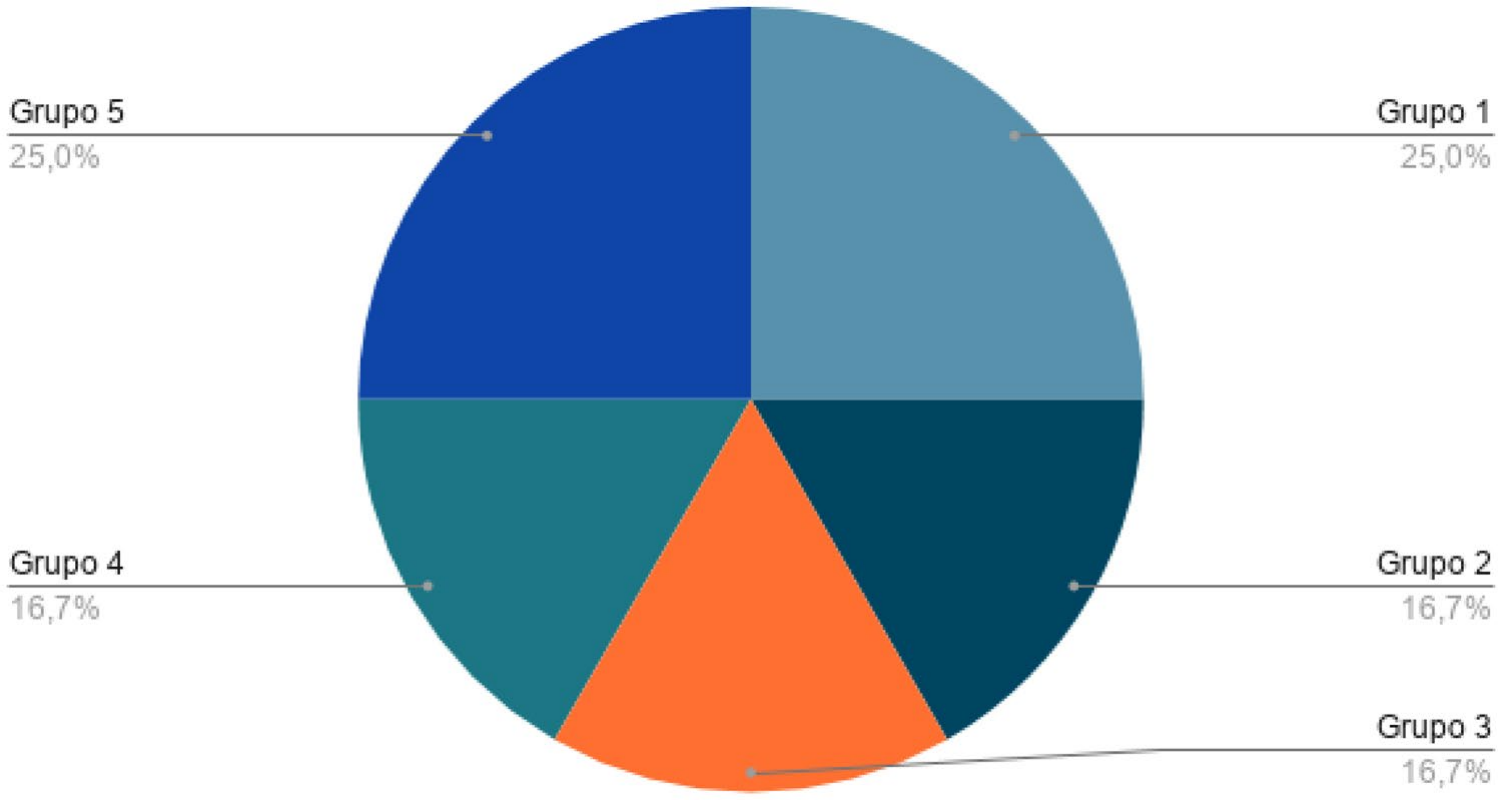
Categorization of apps

Among the health topics most addressed in this review were clinical decisions, as was the case with the literature review, followed by apps for pre-hospital medical care. Consequently, they have been categorised into 5 groups according to the content covered in each of these apps. The following Table 4 and graph in Fig. 7 shows the categories.

**Table 4:** Categorization of apps

Group	Total	References	Percentage
Pre-hospital medical care	3	[44–46]	25%
Applications for COVID-19 management	2	[47, 48]	16.7%
Help with physical illness or disability	2	[49, 50]	16.7%
Searching for clinical material and help among health personnel	2	[51, 52]	16.7%
Clinical decisions	3	[53–55]	25%

**Fig. 8:**
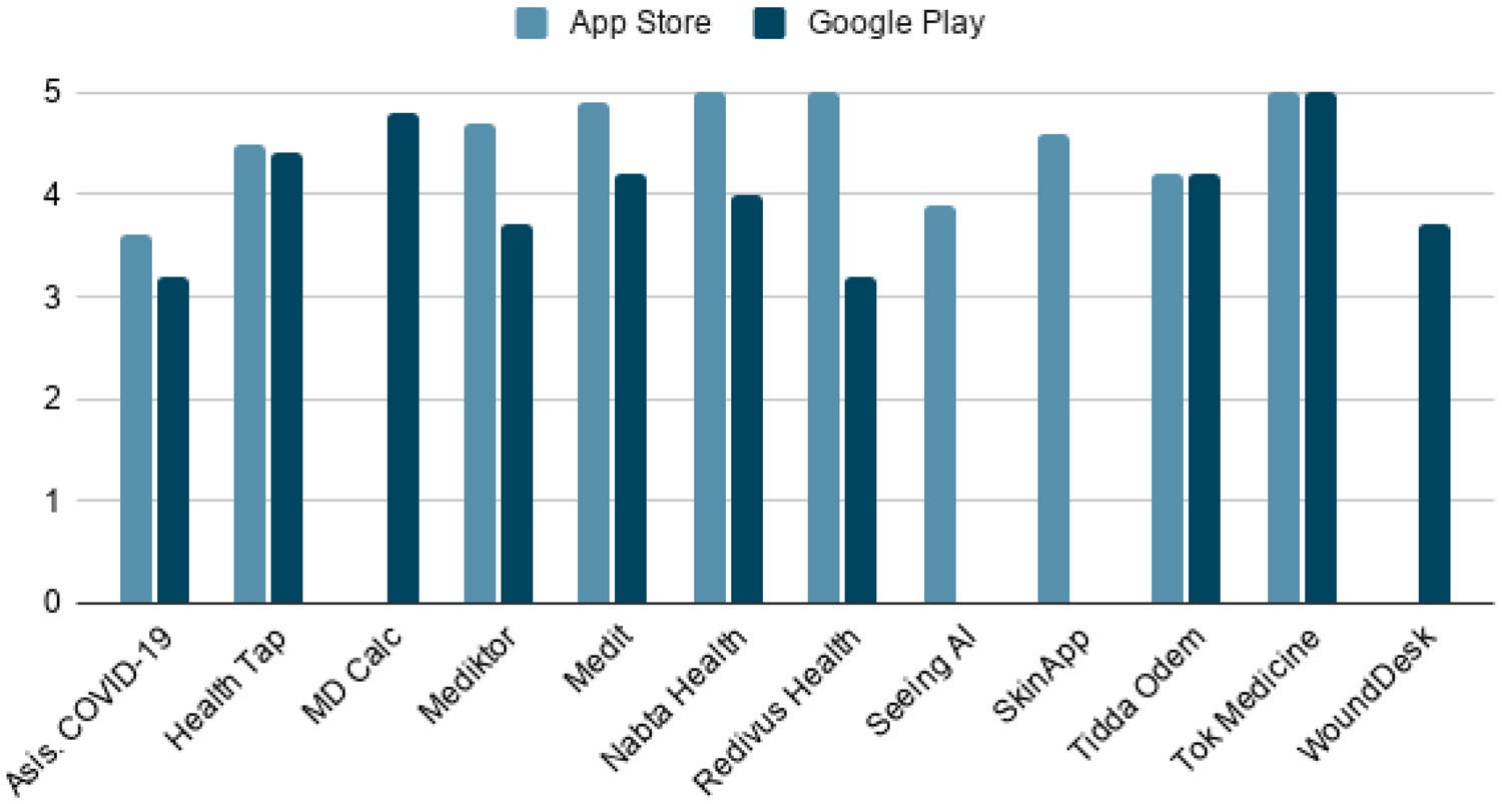
Histogram of ratings for each app

On the other hand, to find out the impact of these apps among users, the ratings of each of them on the two commercial platforms have been studied. Figure 8 below shows graphically the score obtained from 0 to 5 stars.

**Fig. 9:**
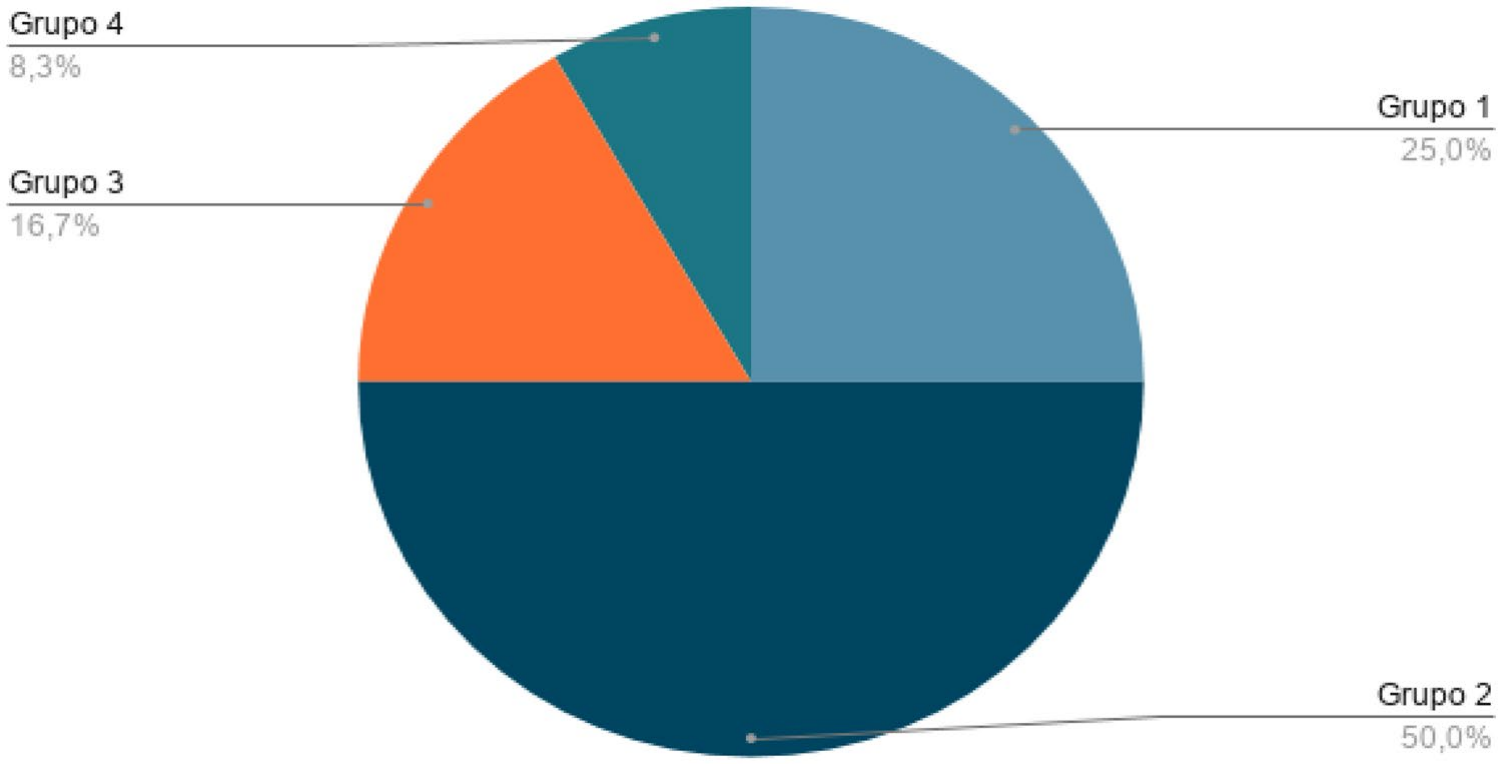
Graph of app technology

**Fig. 10:**
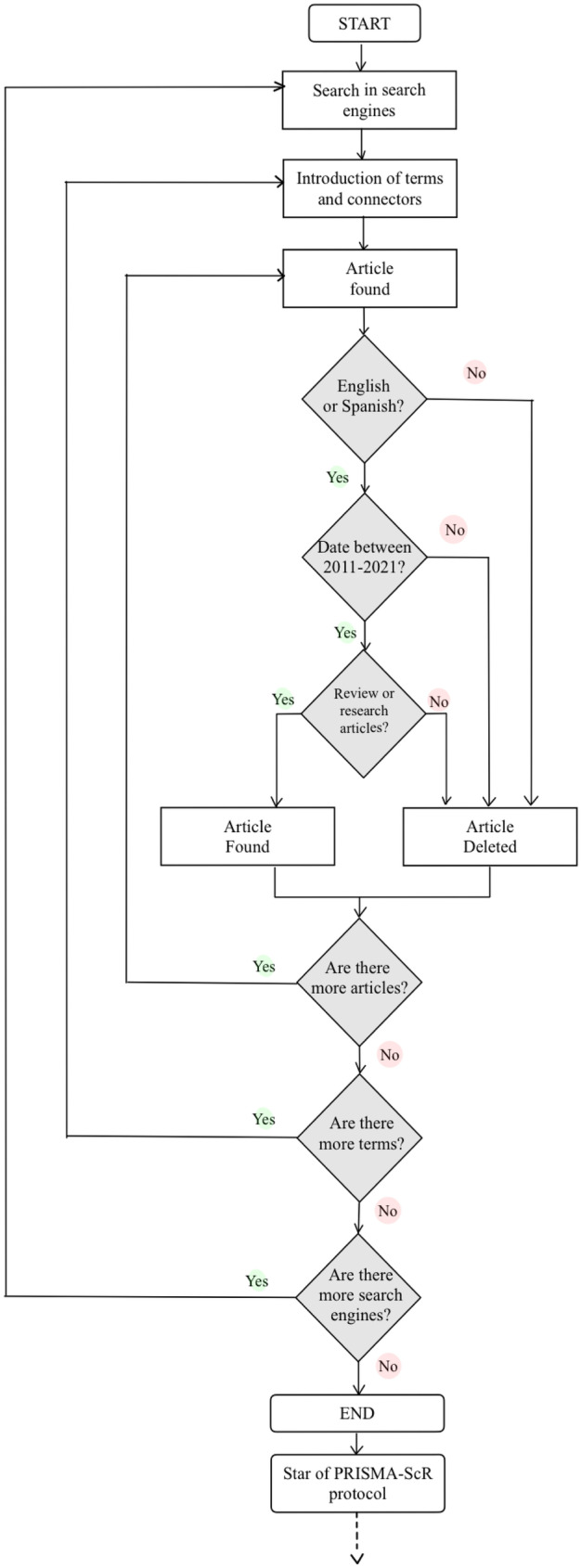
Schematically the procedure used in the search strategy (literature)

**Fig. 11:**
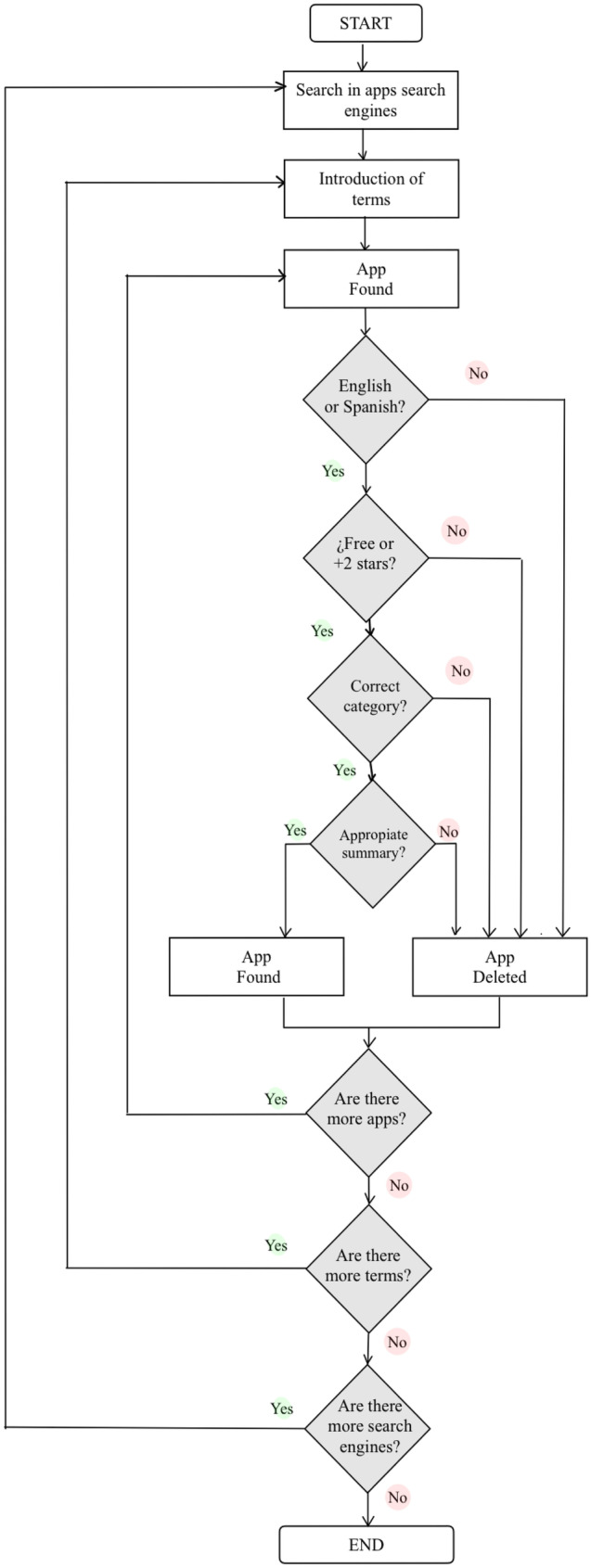
Schematically the procedure used in the search strategy (mobile apps)

The highest-rated app on both platforms is *Tok Medicine*. Followed by *Nabta Health* and *Redivus Health* for App Store users. And *MDCalc* and *HealthTap* for Google Play users. The AI and ML techniques on which each of these tools are based can also be divided into 4 different groups. The following Table 5 and Fig. 9 show the categories.

**Table 5:** Categorization of techniques of apps

Group	Total	References	Percentage
AI-based on evidence	3	[45, 46, 48]	25%
ML-based on natural language processing	6	[44, 47, 48, 51–53]	50%
ML-based on visual processing	2	[50, 54]	16.7%
ML-based on natural language processing + visual processing	1	[49]	8.3%

The most used technique is ML-based on natural language processing, as most apps have a virtual assistant or use an advanced search that allows the user to use tags and filter content. In this way, the system learns automatically according to the user's preferences and over time will be able to suggest recommendations or searches more in line with the user's needs.

### Analysis of the obtained applications

Based on the Core ML [56] model and according to the methodology followed for the selection of each of them, in the following table are 10 apps (out of the 12 existing ones) compatible with the App Store. On the other hand, based on the Kit ML model [57] and the methodology followed for their selection, the following table shows 10 apps (out of the 12 existing ones) compatible with Google Play. The characteristics of the applications included in this systematic review are listed in Appendix C Table 6 [34–45].

## Discussion

A total of 105 scientific articles from the different databases were examined to find the 20 successful research studies. In total, 35% (7/20) were mainly extracted from the Science Direct platform. These studies were further classified into 7 categories, among which 20% (4/20) for medical and/or emergency service research and another 20% (4/20) for automated systems for clinical decisions.

Secondly, during the commercial review, a total of 201 mobile applications were examined and 12 of them were finally chosen. They were classified into 5 groups, of which 25% (3/12) focus on pre-hospital medical care and another 25% (3/12) on clinical decision support. In addition, 50% (6/12) of them use ML techniques based on natural language processing, as most of the apps make use of virtual assistants or advanced searches so that the system subsequently learns from the user's needs.

Contrasting the articles found in the literature review with the applications from the commercial application review, the search for ML-based mobile applications in the literature review leads to low results compared to the commercial review. Therefore, the authors can conclude that the development of mHealth applications is more economically and commercially motivated than research-driven [58].

Mobile apps can optimise the efficiency of health system services, facilitating the work of healthcare staff, and thus reducing their costs. This model can predict emergency admissions to hospitals and help clinicians accurately monitor the risks faced by patients. Establish measures to avoid unplanned admissions that result in high medical costs [59].

Early prediction and reduction of false alarms. Patients will avoid going to the health centre as, by using ML-based mobile health apps, there will be a more informed patient, who will also follow the indications of the treatment to be followed and, therefore medical collapse can be avoided [59]. This is particularly relevant for pandemic scenarios such as COVID-19. In addition, follow-up is improved as information about these patients will be collected and recorded in the electronic medical record, thus improving the intelligent triage system, and assisting physicians in patient triage. Furthermore, mobile health can reduce the workload for medical staff, allowing them to avoid mental saturation that could lead to vital errors.

Nevertheless, several limitations are still to be solved. The lack of availability of technical means in many of the emergency services is a critical barrier. Still, progress is also needed, as more of these studies and trials need to be analysed before a clear conclusion can be drawn on the effectiveness of this machine learning model [59]. As far as the use of mobile applications is concerned, a complete introduction into society is needed based on several functional criteria, such as coordination and uniformity of all systems accessing the data. Another aspect to be highlighted would be the establishment of security criteria regarding the handling and privacy of such data.

As future work, the authors want to explore a machine learning approach to develop a decision support system to support medical emergency scenarios considering the main advantages and limitations of current systems included in this systematic review.

## Conclusions

The emergence of mobile health devices and applications, which can use data and assess a patient's real-time health, lead to a growing trend in machine learning technologies applied to the healthcare industry. The technology can help healthcare experts analyse to identify cases that can lead to improved diagnosis and treatment. On the one hand, this technology will reduce diagnosis times. And speed up immediate patient care. Furthermore, treatments will be optimised and improved. It is expected that, soon, using AI and its subfields, complex algorithmic scoring systems using different variables can be used to predict diagnosis, readmission, and mortality rates. The advantages that machine learning can provide emergency health services play a critical role in improving triage classification and/or diagnosis prediction. Through ML, large-scale analysis is expected to become even more automated than it is today. In the actual pandemic context, the need to analyse data more efficiently arises, whether we are talking about data derived from virus tracking or the analysis of the medical literature that has been generated and collected throughout this review work. The use of AI and ML mechanisms will be the key to efficient global health management [60].

## Appendix C

**Table 6:** Key features of App Store and Google Play apps

Name of The App	Technique	User Type	Health Condition	Search Platform	Language(s)	App Registration?	Category
Asistencia COVID-19 [47]	ML-based on natural language processing	Potential patients	COVID-19	App Store and Google Play	4, including Spanish and English	Yes	Applications for COVID-19 management
HealthTap [45]	AI-based on evidence protocols	Potential patients	Diverse	App Store and Google Play	Spanish and English	Yes, in addition to paying a monthly fee	Pre-hospital medical care
MDCalc [53]	ML-based on natural language processing	Health professional	Clinical decision	Google Play	English	Yes, in addition to paying a monthly fee	Clinical decisions
Mediktor [44]	ML-based on natural language processing	Potential patients	Diverse	App Store and Google Play	9, including Spanish and English	No	Pre-hospital medical care
Medit [51]	ML-based on natural language processing	Health professional	Search for Clinical Material	App Store and Google Play	English	Yes	Searching for clinical material and help among health personnel
Nabta Health [46]	AI-based on evidence protocols	Women patients	Diverse	App Store and Google Play	English and Arabic	Yes	Pre-hospital medical care
Redivus Health [55]	AI-based on evidence protocols	Health professional	Diverse	App Store and Google Play	English	Yes	Clinical decisions
Seeing AI [49]	ML-based on natural language processing + visual processing	Visually impaired patients	Vision loss	App Store	16, including Spanish and English	No	Help with physical illness or disability
SkinApp [50]	ML-based on visual processing	Health professional mainly dermatologists	Dermatology	App Store	French and English	Yes	Help with physical illness or disability
Tidda ODEM [48]	ML-based on natural language processing + visual processing	Health professional Potential patients	COVID-19	App Store and Google Play	German and English	Yes	Applications for COVID-19 management
Tok Medicine [52]	ML-based on natural language processing	Health professional	Search for Clinical Material	App Store and Google Play	Spanish and English	Yes	Searching for clinical material and help among health personnel
+ WoundDesk [54]	ML-based on visual processing	Health professional Patients	Wound Care	Google Play	English	Yes	Clinical decisions

## Abbreviations

AI: Artificial Intelligence
ANN: Artificial Neural Networks
COVID-19: Coronavirus Disease 2019
DL: Deep Learning
EM: Emergency Medicine
ML: Machine Learning
PRISMA-ScR: Preferred Reporting Items for Systematic Reviews and Meta-Analyses Extension for Scoping Reviews
WHO: World Health Organization
